# Effect of Neurosteroids on Basal and Stress-Induced Oxytocin Secretion in Luteal-Phase and Pregnant Sheep

**DOI:** 10.3390/ani13101658

**Published:** 2023-05-17

**Authors:** Patrycja Młotkowska, Elżbieta Marciniak, Anna Misztal, Tomasz Misztal

**Affiliations:** Department of Animal Physiology, The Kielanowski Institute of Animal Physiology and Nutrition, Polish Academy of Sciences, Instytucka 3, 05-110 Jabłonna, Poland

**Keywords:** oxytocin, allopregnanolone, sheep, hypothalamus, pituitary, estrous cycle, pregnant

## Abstract

**Simple Summary:**

Oxytocin (OT) is a hypothalamic hormone that controls key aspects of behavior and female and male reproductive systems as well as playing an important role during the estrous cycle, pregnancy and lactation. Depending on the physiological state, the activity of OT neurons is associated with sex steroid interactions and/or local synthesis of neurosteroids in the brain. This study aimed to determine the central effects of neurosteroids and stress on OT synthesis and release in the hypothalamic-posterior pituitary system in non-pregnant and pregnant sheep. The results showed that allopregnanolone (AL) alone differentially modulated OT synthesis at the hypothalamic and posterior pituitary levels, and markedly inhibited OT secretion induced by stress in non-pregnant sheep. In pregnant animals, an inhibitory effect of neurosteroids on OT secretion was demonstrated after blocking the enzymatic pathway of neurosteroid synthesis. In conclusion, the present study demonstrated the involvement of neurosteroids in the regulation of oxytocin secretion in sheep. The central inhibitory effect of neurosteroids in pregnant sheep is consistent with the concept of protecting the fetus from premature labor.

**Abstract:**

Oxytocin (OT) is a neuropeptide synthesized in the hypothalamic nuclei that modulates both behavioral and reproductive functions, associated with the increased neurosteroid synthesis in the brain. Therefore, the present study tested the hypothesis that manipulation of central neurosteroid levels could affect oxytocin synthesis and release in non-pregnant and pregnant sheep under both basal and stressful conditions. In Experiment 1, luteal-phase sheep were subjected to a series of intracerebroventricular (icv.) infusions of allopregnanolone (AL, 4 × 15 μg/60 μL/30 min) for 3 days. In Experiment 2, pregnant animals (4th month) received a series of infusions of the neurosteroid synthesis blocker, finasteride (4 × 25 μg/60 μL/30 min), conducted for 3 days. In non-pregnant sheep AL alone was shown to differentially modulate OT synthesis in basal conditions, and strongly inhibit OT response to stress (*p* < 0.001). In contrast, in pregnant animals, basal and stress-induced OT secretion was significantly (*p* < 0.001) increased during finasteride infusion compared to controls. In conclusion, we showed that neurosteroids were involved in the control of OT secretion in sheep, particularly under stress and pregnancy conditions and are part of an adaptive mechanism which is responsible for protecting and maintaining pregnancy in harmful situations.

## 1. Introduction

The neuropeptide oxytocin (OT) has important and specific functions in mammals. Studies have shown that it contributes to the transition to motherhood, modulates molecular pathways that suppress the stress response, supports positive mood, and regulates maternal behavior [1]. One of the best-known functions of OT is the crucial role that it plays during labor, i.e., inducing uterine contractions. During lactation, OT is responsible for the release of milk from the mammary glands. Suckling by pups triggers a signal that causes an OT surge from the posterior pituitary (PP) into the circulation, which in turn stimulates the myoepithelial cells surrounding the mammary gland follicles to contract, effectively expelling milk through larger ducts [2,3]. In addition to its peripheral functions, OT also exerts effects on the central nervous system (CNS), influencing the reproductive process and social behavior, including sexual, maternal, and mating behavior in monogamous species [4].

The neurons that synthesize OT are located in the supraoptic (SON) and paraventricular (PVN)—paired nuclei of the hypothalamus that project their axons to the PP, where OT is released into the bloodstream [5]. OT is initially synthesized as an inactive precursor protein encoded by the OT gene. Subsequently, it is hydrolyzed into smaller fragments through a cascade of enzymes with a peptidylglycine α-amidating monooxygenase (PAM) being a key enzyme in this process. PAM is an integral membrane bifunctional protein expressed in a wide variety of cell types, including endocrine, neuronal, glial, and endothelial cells, where it catalyzes the final reaction in the maturation of α-amidated peptide hormones [6]. In peripheral and CNS neurons, PAM is localized within the large dense-core vesicles in axons and nerve terminals [7,8]. Animals that lack the fully active form of the enzyme exhibit a wide range of disorders, including altered inhibitory synaptic neurotransmission, increased anxiety-like behavior, and problems with maintaining body temperature in cold environments [6,9,10]. OT is also released from the dendrites and somas of OT neurons within the hypothalamus, as well as from axon terminals in intracerebral target regions, such as the medial amygdala [11]. Together with the closely related arginine vasopressin (AVP), the two hormones essentially form the hypothalamic-neuro-hypophyseal system in mammals. Moreover, OT can act through both OT and vasopressin receptors [12].

Stressful stimuli trigger numerous responses in the body, including the release of OT into the circulation and locally within the brain [13,14,15,16]. OT was shown to reduce the stress-induced response of the hypothalamic-pituitary-adrenal (HPA) axis as well as sympathetic activity and anxiety-related behaviors in laboratory animals and humans [17,18]. Activation of the OT system is thought to be an adaptive strategy when faced with stressful or threatening situations. Interestingly, the activation of OT neurons through social support also reduces stress responses [19]. According to Papadimitriou and Priftis (2009), central OT signaling acts as a buffer against stressful events and experiences, thereby reducing susceptibility to psychopathology and physical diseases [20].

Neurosteroids, which are produced locally in the CNS, are another important factor involved in the stress response [21,22]. It has been shown that both corticotropin-releasing hormone (CRH) and adrenocorticotropic hormone (ACTH), the main components of the HPA axis, can activate the biosynthesis of neurosteroids in the brain. The most recognized neurosteroid is allopregnanolone (AL), which is synthesized locally from cholesterol or produced by the conversion of progesterone by a set of enzymes: 5α-reductase and 3α-hydroxysteroid dehydrogenase (3α-HSD) [23]. Locally produced AL may control numerous neuronal functions, especially HPA axis activity. Elevated brain concentration of AL was demonstrated to potentiate the stress-suppressive effects of γ-aminobutyric acid (GABA) by binding to inhibitory membrane-bound GABA_A_ receptors in the PVN [23]. Interestingly, activation of GABA_A_ receptors also exhibited also a tonic inhibitory influence upon OT release in vivo, and AL helped inhibit OT neurons in vitro [24].

Our previous study demonstrated the anti-stress effect of AL in sheep in relation to the HPA axis [25]. We also observed an increase in cerebrospinal fluid (CSF) AL concentration in pregnant sheep up to the fifth month of pregnancy, indicating the potential involvement of neurosteroids in the regulation of physiological processes during pregnancy [26]. Building on these findings and considering the role of OT in both stress response and pregnancy physiology, we aimed in the present study to test the hypothesis that manipulating central neurosteroid levels could affect OT synthesis and release in non-pregnant (luteal-phase) and pregnant sheep under both basal and stressful conditions.

## 2. Materials and Methods

### 2.1. Animal Handling

Thirty-six Polish Longwool sheep, aged 2 years and weighing 55 ± 3 kg, were used in two experiments. This breed of sheep displays seasonality in their reproduction. The animals were housed indoors in individual pens with natural light at a location of 52° N, 21° E. They were fed twice daily a diet based on pellet concentrate that followed the recommendations of the National Research Institute of Animal Production in Krakow–Balice, Poland, and the National Institute for Agricultural Research in France [27]. Pregnant sheep were provided with a balanced diet that met their daily nutrient requirements, which included a dry matter intake of 1.4–1.8 kg, crude protein intake of 160–300 g, and net energy intake of 6.5–10.5 MJ. All sheep had ad libitum access to hay, water, and mineral licks.

### 2.2. Brain Surgeries

One month prior to the start of experiments, the sheep underwent brain surgery, in which a stainless-steel guide cannula was implanted into the third ventricle (IIIv) of the brain (outer diameter 1.2 mm, frontal positions: 30.5–31.5 mm, sagittal position: 1.0 mm), using the stereotaxic coordinate system for the hypothalamus of the sheep [28]. Implantation was conducted under general anesthesia (xylazine: 40 mg/kg of body weight, intravenously; xylapan and ketamine: 10 to 20 mg/kg of body weight, intravenously; Bioketan, Vetoquinol Biowet, Pulawy, Poland) through an orifice bored in the skull, following the protocol described by Traczyk and Przekop [29]. The guide cannula was secured to the skull with stainless steel screws and dental cement, and the external orifice of the canal was sealed with a stainless steel lid. After the procedure, the sheep received daily antibiotics for 5 days (1 g streptomycin and 1,200,000 IU benzylpenicillin; Polfa, Warsaw, Poland) and analgesics injections for 4 days (metamizole sodium 50 mg/sheep; Biovetalgin, Biowet Drwalew, Poland, or meloxicam 1.5 mg/sheep; Metacam, Boehringer Ingelheim, Ingelheim am Rhein, Germany). Cannula placement in the 3rd ventricle was confirmed by CSF efflux during procedure and after slaughter and data herein come from only animals with correct cannula placement.

### 2.3. Procedures and Design of Experiment 1: Sheep in the Luteal Phase

In Experiment 1, 24 reproductively active sheep were used from mid-October to mid-December. Estrus synchronization was achieved through the Chronogest-CR technique as previously described [25,26]. The sheep were randomly assigned to one of four groups (n = 6) and were subjected to the following treatments for 3 consecutive days of the late luteal phase (days 12–14) of the estrous cycle: (i) intracerebroventricular (icv.) vehicle infusion for 3 days (C1), (ii). icv. infusion of AL (Sigma-Aldrich, Saint Luis, MO, USA) alone for 3 days (A); (iii) icv. vehicle infusion for 3 days, and subjected to stress stimuli on day 3 (S1); and (iv) icv. AL infusion for 3 days and application of stress stimuli on day 3 (AS). AL dose (60 μg/day), was administered based our preliminary study (unpublished data, Grant No. 2015/19/B/NZ9/03706). AL was reconstituted in a solution of DMSO (Blirt, DNA-Gdańsk, Gdańsk, Poland) and 20% 2-hydroxypropyl-β-cyclodextrin (1:1) (Sigma-Aldrich, Saint Luis, MO, USA) and subsequently diluted using Ringer-Locke (RL) solution [25]. The control vehicle, without AL, was also diluted in RL and used for infusion.

All infusions were conducted using a BAS Bee microinjection pump and calibrated 1.0-mL gas-tight syringes, through a series of four 30-min infusions, at 30-min intervals (AL dose: 4 × 15 μg/60 μL/30 min) from 10:00 to 14:00. Intermittent infusion of the active substance into the CNS was previously validated in our sheep model [25,26] as maintaining effective signaling without completely blocking the receptors and/or binding sites. During the trial, the sheep were kept in pairs in the experimental room in comfortable cages, where they had been previously adapted for at least 2 days and could lie down. After the first blood sample collection, stress stimuli, including isolation (removal of untreated sheep) and partial restriction of movement preventing escaping from the cage, were applied for the duration of the infusion period (4 h). The combination of both stressors was validated previously in cycling, pregnant and lactating sheep [25,30,31], demonstrating a clear response of the HPA axis at the level of all its components. During the 4-h experiment on the third day, blood was sampled via a jugular vein catheter that had been inserted a day prior to the collection. A total of 125 mL of blood was drawn, with 5 mL taken every 10 min to a prechilled tubes containing EDTA (50 mmol) and aprotinin (2.4 trypsin inhibitor units; Sigma–Aldrich, St. Louis, MO, USA), centrifuged, and then plasma was stored at −20 °C until OT was assayed. Directly after the experiment, the animals were pharmacologically stunned with xylazine (0.2 mg/kg body weight) and ketamine (3 mg/kg body weight) administered intravenously, and subsequently euthanized. The brains, along with the pituitaries, were then rapidly removed from the skull. After separating the median eminence (ME), the brains were sectioned sagittally into cerebral hemispheres. The extracted blocks of the anterior hypothalamus (cut to a depth of 2 mm) were divided into two parts: one part containing the paraventricular nucleus (PVN) and supraoptic nucleus (SON), according to the stereotaxic atlas of the ovine hypothalamus [28]; additionally, PP tissue was collected. All tissue cutting procedures were carried out on sterile glass plates on ice, and the extracted structures were immediately frozen in liquid nitrogen and stored at −80 °C until analysis.

### 2.4. Procedures and Design of Experiment 2: Pregnant Sheep

For Experiment 2, 12 pregnant sheep at 16 weeks of gestation with high AL levels in the cerebrospinal fluid [26] were used. After one month of cannula placement, the sheep were randomly assigned to two groups (n = 6) and received the following treatments: (i) icv. vehicle infusion for 3 days (C2), and subsequent icv. finasteride (Sigma-Aldrich, Saint Luis, MO, USA) infusion for 3 days (F), one week apart, and (ii) icv. vehicle infusion for 3 days and introduction of stress stimuli on day 3 (S2), followed by icv. finasteride infusion for 3 days and introduction of stress stimuli on day 3 (SF), one week apart. Finasteride, which is the neurosteroid synthesis blocker, was dissolved in a mixture (1:1) of DMSO (Blirt, DNA-Gdańsk, Gdańsk, Poland) and 20% 2-hydroxypropyl-β-cyclodextrin (Sigma-Aldrich, Saint Luis, MO, USA). After 24 h, Ringer-Locke (RL) solution was added to the mixture and thus prepared drug was aliquoted for all sheep. The solution without finasteride was diluted in RL and applied as a control vehicle. Each aliquot of the infusion mixture contained 2% DMSO. Infusions were carried out in a series of four 30-min infusions, at 30-min intervals (finasteride dose: 4 × 25 μg/60 μL/30 min) as describe in the experiment 1. The dose of finasteride was chosen on the basis of the rodent research literature [32] and our preliminary study. Five milliliters of blood were taken every 10 min (on the 3rd day, total 125 mL) to prechilled tubes with EDTA (50 mmol) and aprotinin (2.4 trypsin inhibitor units; Sigma–Aldrich, St. Louis, MO, USA), centrifuged, and plasma was subsequently stored at −20 °C until OT was assayed.

### 2.5. Analysis of Gene Expression Levels: RNA Isolation, cDNA Synthesis and Quantitative Real-Time Polymerase Chain Reaction (RT-qPCR)

Total RNA was isolated from the hypothalamus with the NucleoSpin RNA II kit (MACHEREY-NAGEL GmbH and Co., Düren, Germany) following the manufacturer’s instructions. The quality and quantity of RNA were determined using a NanoDrop ND-1000 spectrophotometer (Thermo Fisher Scientific, Waltham, MA, USA). Complementary DNA (cDNA) was synthesized using a TranScriba Kit (A&A Biotechnology, Gdynia, Poland) with 1 µg of total RNA in a 20-µL reaction volume. The real-time PCR reactions were conducted using 5 × HOT FIREPol^®^ EvaGreen qPCR Mix Plus (Solis BioDyne, Tartu, Estonia) with 2 µL of cDNA template, 1 µL of primers (0.5 µL each, 10 pmol/mL), 3 µL of PCR Master Mix buffer, and 9 µL of ddH_2_O. The following PCR reaction conditions were applied: initial denaturation step: 95 °C, 15 min, denaturation step: 95 °C, 15 s, annealing step: 60 °C, 20 s, and elongation step: 72 °C, 20 s (40 cycles). Primers were designed (Table 1) using Primer3 software (The Whitehead Institute, Boston, MA, USA) to analyze the following genes of interest: *OT (OT)* and peptidylglycine a-amidating monooxygenase *(PAM)* in the hypothalamus and pituitary, and genes that served as endogenous controls: glyceraldehyde-3-phosphate dehydrogenase (*GAPDH*) and peptidylprolyl isomerase C (*PPIC*). Amplification specificity was additionally validated by electrophoresis of the obtained products in a 2% agarose gel and visualized using a camera with UV transilluminator.

The Rotor Gene 6000 v. 1.7 software (Qiagen, Hilden, Germany) was used to analyze the data, applying the comparative quantification option, and the Relative Expression Software Tool (2008), as described by Pfaffl et al. [33], on the basis of a correction algorithm of PCR efficiency developed by Pfaffl et al. [34]. Gene expression levels were normalized by calculating the geometric means of two reference genes, i.e., *GAPDH* and *PPIC*, which were determined in all samples to account for variations in cDNA concentration and PCR efficiency between tubes.

### 2.6. Hormone Concentration Assay

Plasma OT levels were determined using the double-antibody radioimmunoassay method with an n OT RIA kit RK-051-01 (Phoenix Pharmaceuticals Inc., Burlingame, CA, USA), following the producer’s protocol. This method was previously validated in sheep in a study of Górski et al. [35]. The sensitivity of the test was 1 pg/mL, and the variability within and between tests was 9% and 15%, respectively.

### 2.7. Statistical Analyses

The results of gene expression and hormone levels are reported as means ± SEM. The normality of all data was assessed using the Shapiro-Wilk normality test and grouped into parametric and nonparametric data sets. The levels of OT in plasma were evaluated at 1-h intervals by repeated measures analysis of variance (STATISTICA, Stat Soft, Tulsa, OK, USA), where time and treatment served as repeated measurement factors. After each analysis, a post-hoc test of least significant difference was performed. Samples that were used for baseline value calculations in both experiments were taken before treatments (at 10:00 a.m.) and analyzed separately using the non-parametric Kruskal-Wallis test. Statistical evaluations of differences in OT and PAM genes expression were carried out using non-parametric statistics: the Kruskal–Wallis test with multiple comparisons of average ranks and then the Mann–Whitney *U* test for individual groups.

## 3. Results

### 3.1. Experiment 1: Sheep in the Luteal Phase

#### 3.1.1. OT and PAM mRNA Expression in the Hypothalamic Nuclei

**Figure 1:**
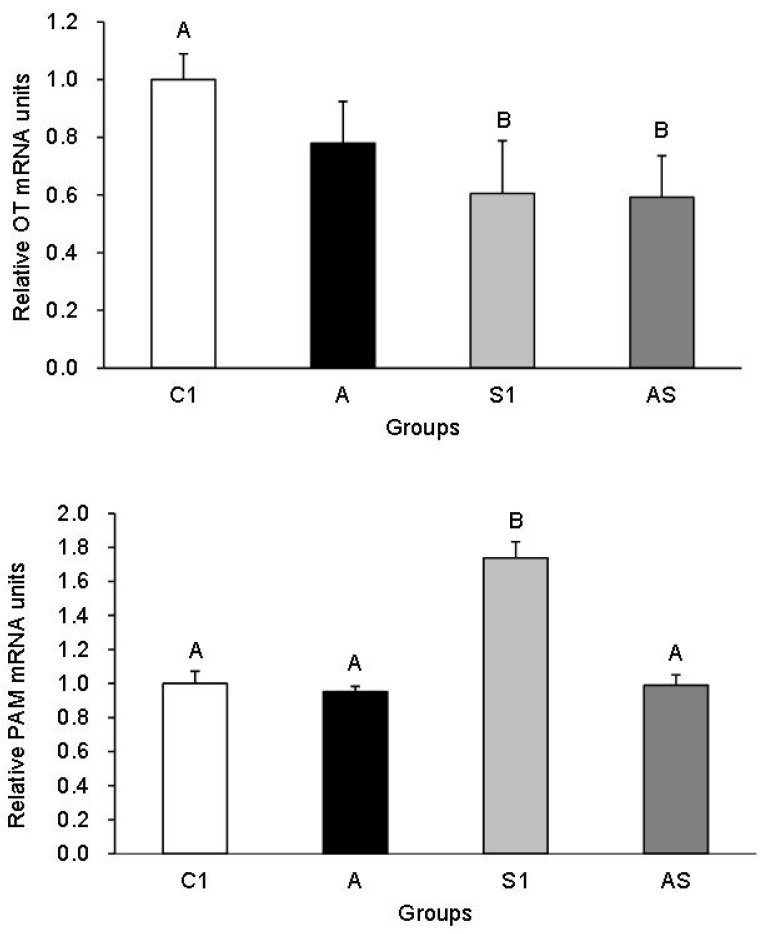
Relative mRNA expression (mean ± SEM) of oxytocin (OT) (**top**) and peptidylglycine a-amidating monooxygenase (PAM) (**bottom**) in the paraventricular nuclei of sheep treated with: vehicle (C1), allopregnanolone alone (A), vehicle and stressful stimuli (S1) and allopregnanolone with stressful stimuli (AS). Statistical significance was determined at the following levels: AB, *p* < 0.01.

The data obtained from real-time qPCR analyses showed that both *OT* and *PAM* transcripts were expressed in the PVN and SON hypothalamic nuclei. Figure 1 displays the relative abundance of *OT* and *PAM* mRNA in the PVN of sheep from all experimental groups. The relative abundance of OT mRNA decreased in sheep from Groups S1 (*p* < 0.01) and AS (*p* < 0.01) compared to controls. In contrast, there was an increase in PAM transcript levels in Group S1 compared to the control and the other groups (*p* < 0.01). Interestingly, AL treatment reduced PAM mRNA expression level in Group AS (*p* < 0.01) compared to that of the Group S1, but AL alone had no effect on the relative abundance of OT nor PAM transcripts in the PVN compared to controls.

**Figure 2:**
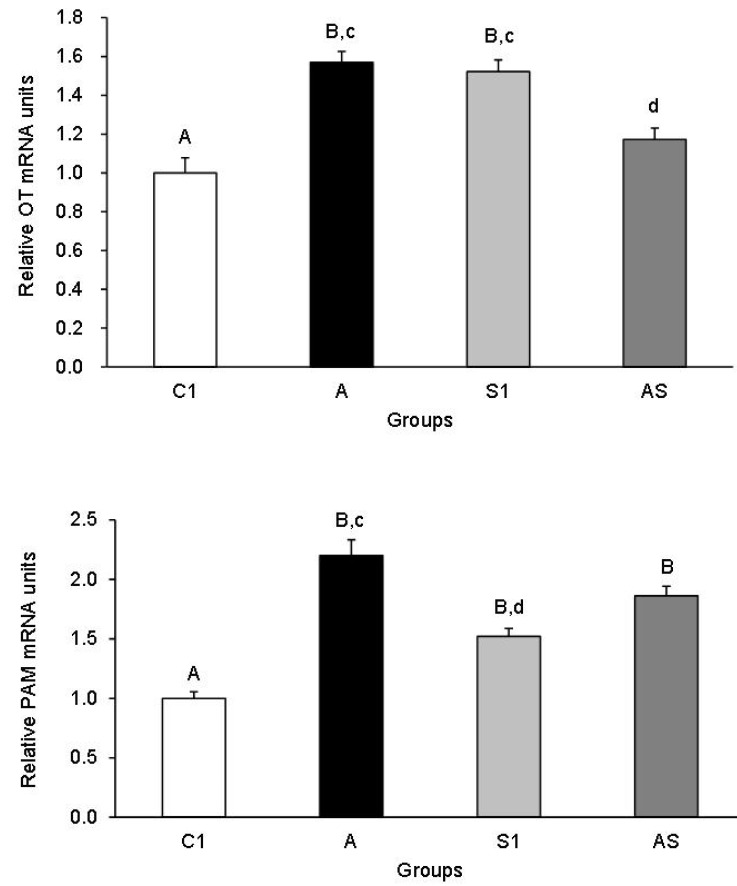
Relative mRNA expression (mean ± SEM) of oxytocin (OT) (**top**) and peptidylglycine a-amidating monooxygenase (PAM) (**bottom**) in the supraoptic nucleus of sheep treated with: vehicle (C1), allopregnanolone alone (A), vehicle and stressful stimuli (S1) and allopregnanolone with stressful stimuli (AS). Statistical significance was determined at the following levels: cd, *p* < 0.05 and AB, *p* < 0.01.

The relative abundance of OT and PAM mRNA in the SON of sheep from all treatment groups is shown in Figure 2. Both AL alone (Group A) and stressful stimuli (Group S1) increased the OT mRNA abundance compared to the control group (*p* < 0.01). However, AL lowered OT transcript level in Group AS in comparison to Groups A and S1 (*p* < 0.05). The relative abundance of PAM mRNA increased in all treatment groups compared to the control group (*p* < 0.01). In addition, the abundance of PAM mRNA in Group A was also higher (*p* < 0.05) compared to Group S1.

#### 3.1.2. OT and PAM mRNA Expression in the PP

**Figure 3:**
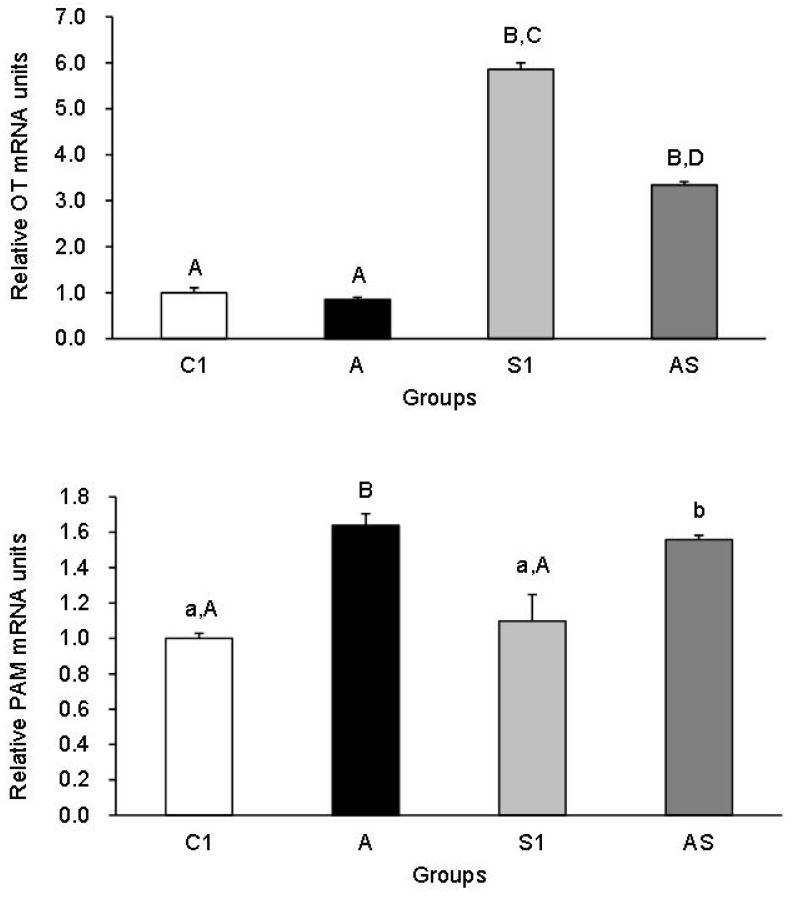
Relative mRNA expression (mean ± SEM) of oxytocin (OT) (**top**) and peptidylglycine a-amidating monooxygenase (PAM) (**bottom**) in the posterior pituitary of sheep treated with: vehicle (C1), allopregnanolone alone (A), vehicle and stressful stimuli (S1) and allopregnanolone with stressful stimuli (AS). Statistical significance was determined at the following levels: ab, *p* < 0.05, AB, *p* < 0.01 and CD, *p* < 0.001.

Figure 3 illustrates the proportionate presence of OT and PAM mRNA in the PP of sheep from all experimental groups. The expression of OT mRNA increased in Groups S1 and AS (*p* < 0.01), while it was at a similar level in Group A compared to the control. The level of OT gene transcript in Group S1 was also higher than in Groups AS and A (*p* < 0.01), and that in Group AS was higher than in Group A (*p* < 0.01). Regarding the PAM transcript level, increases (*p* < 0.05–*p* < 0.01) were recorded in Groups A and AS in relation to Groups C1 and S1.

#### 3.1.3. Plasma OT Concentration

Mean plasma OT concentrations in the first samples collected before the start of infusion and stress (10:00 a.m.) did not differ between the groups of luteal-phase sheep (data not shown).

**Figure 4:**
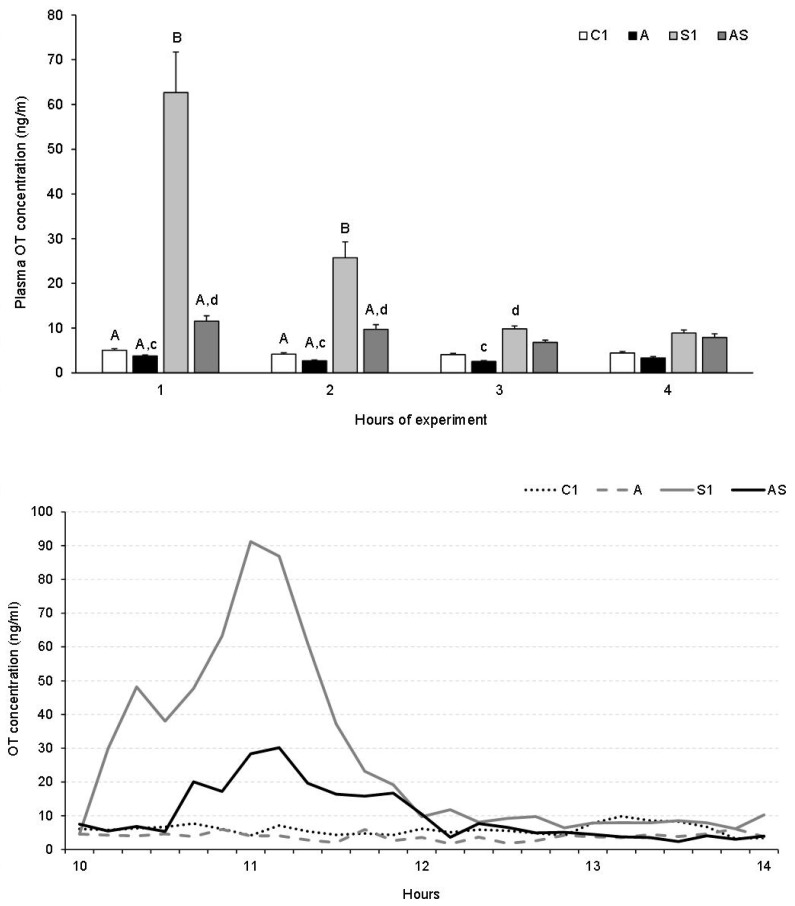
Plasma oxytocin (OT) levels (mean ± SEM) by 1-h consecutive experimental periods (**top**) and OT time-concentration profiles (**bottom**) in representative sheep treated with: vehicle (C1), allopregnanolone alone (A), vehicle and stressful stimuli (S1) and allopregnanolone with stressful stimuli (AS). Statistical significance was determined at the following levels: cd, *p* < 0.05 and AB, *p* < 0.001.

The analysis of mean OT concentrations according to 1-h periods revealed an increase (*p* < 0.001) in OT level in Group S1 in the first and second hour of the experiment compared to the control and other Groups (Figure 4 top). During this period, a significant decrease in OT concentration in Group AS was observed (*p* < 0.001) in response to AL infusion compared to Group S1. AL alone had no significant effect on plasma OT concentration compared to control animals, but OT concentration in Group A was lower than those in Groups S and/or AS (*p* < 0.05 and *p* < 0.001) during the first, second and the third hour. There were no statistical differences in OT levels between groups in the fourth hour of the experiment. The time-concentration profiles of OT in individual representative sheep from all treatment groups in Experiment 1 are shown in Figure 4 (bottom).

### 3.2. Experiment 2: Pregnant Sheep

#### Plasma OT Concentration

**Figure 5:**
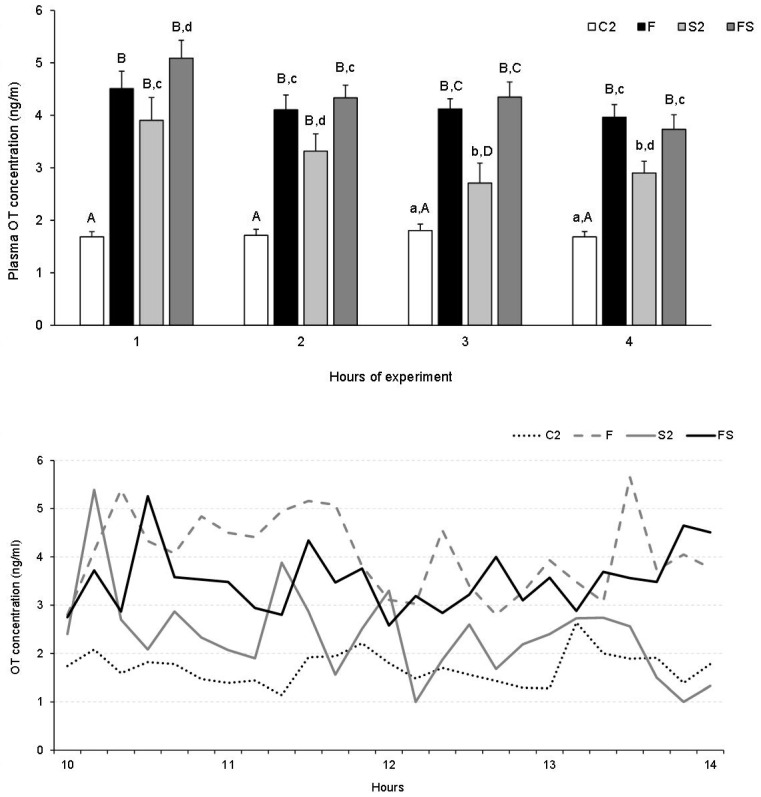
Plasma oxytocin (OT) levels (mean ± SEM) according to 1-h consecutive experimental periods (**top**) and OT time-concentration profiles (**bottom**) in representative sheep treated with: vehicle (C2), finasteride alone (F), vehicle and stressful stimuli (S2) and finasteride with stressful stimuli (FS). Statistical significance was determined at the following levels: Significance of differences: ab and cd, *p* < 0.01 and AB and CD *p* < 0.001.

There were no significant differences in the average plasma OT levels of pregnant sheep groups in the first samples collected before the initiation of infusion and stress at 10:00 a.m. (data not shown). The analysis of mean plasma OT levels by 1-h periods showed that in sheep treated with finasteride (Group F), stress (Group S2), and stress in combination with finasteride (Group FS), these levels increased rapidly (*p* < 0.01–*p* < 0.001) and remained high until the end of the experiment compared to the control group (Figure 5 top). Furthermore, plasma OT levels in sheep from Groups F and FS were higher (*p* < 0.01–*p* < 0.001) than in sheep from Group S2 throughout the experiment. The time-concentration profiles of OT in representative sheep from all treatment groups of Experiment 2 are shown in Figure 5 (bottom).

## 4. Discussion

The current study’s findings indicate the specific role of neurosteroids in regulating the secretion of OT in cyclic (luteal-phase) and pregnant sheep under both basal and stressful conditions. In the luteal phase-sheep, the expression of the OT and PAM genes was examined in the hypothalamus and PP, and OT concentration in the systemic circulation.

OT secretion largely depends on the physiological state of the animal. Its biosynthesis can be regulated by the sex hormone interactions as the OT gene contains an estrogen receptor—responsive element [36]. Early studies in rats showed that declining progesterone levels in estradiol-primed animals increased hypothalamic OT expression, while retaining high progesterone concentration attenuated this effect [37]. Changes in the brain OTergic neurons through not only known nuclear receptors and their direct genomic actions, but also membrane steroid receptors and steroid-binding globulins and their putative receptors [38]. To avoid fluctuations in estrogen levels in cycling animals, our study used AL in late luteal-phase sheep, whose reproductive status was previously described by Młotkowska et al. [39]. Early studies in sheep showed that circulating OT concentrations were similar to those of progesterone during the estrous cycle and reached low levels during the period of luteal regression [40,41].

Under basal conditions, generally no changes were observed in OT gene expression or OT release into the bloodstream after AL administration. The lack of a clear response of the OT and PAM genes to AL in the PVN could indicate the absence of an active interaction between neurosteroids and OT in regulating CRH neurons under basal conditions. On the other hand, AL is a potent allosteric modulator of GABA_A_ receptors, where it strengthens the inhibitory effect of GABA [42]. Thus, due to the inhibitory GABAergic neurotransmission, the expression of the OT gene in the PVN may also be unaffected. The only stimulatory effect of AL on OT mRNA expression was observed in the SON. In addition, AL stimulated PAM gene expression in the SON and in the PP, indicating a diverse response of hypothalamic OT neurons to neurosteroids in terms of hormone synthesis. Previous research has shown that in young rats, large amounts of OT were released from the somato-dendritic compartments of SON neurons in response to a variety of neurosteroids, while in adult animals, the Al-induced OT release was much smaller [43]. Although the exact mechanism of OT response in young rats is related to specific GABAergic activity during development, this remains to be fully elucidated in sheep.

The present study also demonstrated different changes at the hypothalamic and pituitary levels in the process of OT synthesis in luteal-phase sheep in response to acute stress stimuli. OT mRNA levels decreased in the PVN, while they increased in the SON and PP. Notably, OT mRNA levels in the PP were almost four times higher than in the SON, and ten times higher than in the PVN. This finding suggested that, in addition to the mature hormone, significant amounts of OT mRNA were also translocated from both hypothalamic nuclei to nerve terminals in the PP. The phenomenon of axonal transport has been described previously for hypothalamic vasopressin neurons [44], as well as for GnRH neurons [45]. According to Kaplan et al. [46], biosynthesis of peripheral protein does not occur during basal conditions, but it is initiated in response to external stimuli. Therefore, the observed increase in axonal transport of OT mRNA in stressed animals could be particularly important in increasing the pool of OT transcript for hormone synthesis also in the vicinity of the release site. On the other hand, the marked stimulation of PAM mRNA expression and presumably enzyme activity in the PVN could suggest elevated OT mRNA translation and hormone synthesis under stress conditions. This response may have been triggered by a stress-induced OT surge into the bloodstream, which was observed in the stressed sheep during the first two hours of the experiment. Specific changes in the OT synthesis process at the hypothalamic and PP levels may reflect the need for the hormone in a given situation. The elevated levels of OT expression and release after exposure to various of stressors, including restraint, immobilization, foot shock and forced swimming have been demonstrated in numerous rodent studies [47,48,49]. Stimulation of OT release, like the HPA axis activity, involves a number of complex mechanisms dependent on a wide range of physical and psychological stressors. Under stress conditions, OT neurons may be partially activated by A2 noradrenergic/prolactin-release peptide (PrRP) neurons in the nucleus of the solitary tract in the medulla oblongata and neurons of the medial amygdala. The activation of OT neurons by noxious stimuli has also been suggested to be mediated by A1 noradrenergic neurons in the medulla oblongata [50,51]. Vacher et al. [52] showed that OT neurons in the PVN could be spontaneously modulated by administering norepinephrine, which stimulated the expression of the OT gene in both the PVN and SON. OT neurons located in the PVN are also innervated by hypothalamic dopaminergic neurons, and dopaminergic receptors D2, D3 and D4 have been found in their cell bodies [53,54]. Interestingly, a reciprocal activation between the OT system and HPA axis, indicating bidirectional regulation, has been suggested. In response to stress, OT release from PVN magnocellular neurons is mediated by the binding of CRH to the CRF2 receptors on OTergic neurons of the PVN. Subsequently, the released hormone acts on its receptors in many neural structures in the brain, including PVN cells that produce CRH with increased activity during stress. In the latter case, local dendritic release of OT in the PVN may affect the activity of PVN CRH neurons during acute stress [55], leading to downregulation of CRH gene expression and inhibition of stress-induced activity of the HPA axis [56,57]. In addition, OT both regulates and is regulated by glucocorticoids (GC) as it decreases stress-induced GC levels, while corticosterone increases OT levels in most cases. In female rats, subcutaneous OT administration was shown to reduce the expression of glucocorticoid receptors in the hippocampus and decrease the effect of corticosterone in plasma [58]. On the other hand, the absence of glucocorticoids, resulted in a reduced OT binding to its receptors in the hippocampus of male rats and an abolition of stress-induced OT release during forced swimming [59]. Despite this, it is generally accepted that the released OT inhibits stress axis activation. By acting on other brain structures, such as hippocampus, amygdala, bed nuclei of the stria terminalis, ventrolateral septum, and several hypothalamic nuclei, which express OT receptors, the released hormone may induce an inhibitory modulation over the HPA axis by their GABAergic efferents to the PVN [60]. Intranasal or intraventricular administration of OT was shown to attenuate the response of the HPA axis to acute stress, while intracerebral administration of an OT receptors antagonist increased both basal and stress-induced HPA axis activity [61,62]. Moreover, exogenous OT was found to reduce stress-induced CRH mRNA expression in the PVN in OT knockout mice [63].

Numerous animal studies have focused on the role of neurosteroids during stress conditions, because their action is associated with the “tuning” of inhibitory GABAergic transmission in the CNS [64]. In the present study, we observed an intriguing OT response following central AL infusion in stressed sheep. AL inhibited stress-induced OT mRNA expression in the SON and PP but stimulated PAM mRNA expression, similarly to when administered alone. Despite the increased expression of the enzyme, the observed decrease in OT mRNA levels may have been due to the inhibitory effect of AL rather than the increased translation of hormone protein, since stress-induced OT release into the circulation was also strongly reduced. In this case, there could be a direct involvement of GABAergic transmission as potentiated by AL at the hypothalamic and/or PP level [47]. In addition, OT neurons could be inhibited by opioids; however, opioidergic inhibitory tone was shown to predominate during pregnancy [65,66]. The neurosecretory OT response to stressful stimuli was similarly reduced by AL, as was observed for the HPA axis in our previous study [25]. This indicated that the neuroendocrine stress response was inhibited in all compartments of the HPA axis. Specifically, AL reduced the expression of CRH and vasopressin (AVP) mRNA in the PVN, as well as AVP receptor (V1b) and POMC transcript levels in the anterior pituitary and plasma ACTH and cortisol levels in stressed sheep. It should be noted that OT itself may be involved in suppressing the HPA axis response to stress. Studies have shown that OTergic neurons in the PVN are able to affect CRH and AVP neurons in sheep [67].

The intriguingly low plasma OT concentrations observed in our pregnant sheep confirmed several early observations. OT levels were shown to remain low throughout pregnancy until term, i.e., at concentrations even below those observed during the luteal phase of the cycle [40,68]. However, episodic increases in OT concentration were found several days after mating [69]. Importantly, the role of OT during the first phase of pregnancy is specifically targeted at maintaining it. The hormone is involved in the differentiation of placental cells and steroidogenesis, but its secretion seems to be steroid-dependent [70]. Russell et al. [71] demonstrated that OT accumulation in the PP was increased at the end of pregnancy, mainly as a result of decreased release, providing for the large stimulated increase in OT secretion during parturition. A sudden surge in OT due to unexpected stimulus during pregnancy could lead to uterine contractions and preterm labor. Although progesterone is widely recognized to play a major role in maintaining uterine quiescence during pregnancy [72], its metabolite AL is believed to have a stronger effect in the CNS [67]. We have previously demonstrated that pregnancy in sheep is associated with a temporal increase in brain AL concentration [26]. In the present study, the central treatment of late-pregnancy sheep with a neurosteroid synthesis inhibitor, finasteride, led to an increase in OT release. In addition, the applied regimen of serial finasteride infusions maintained high plasma OT levels throughout the experiment. Importantly, stressed sheep had significantly higher levels of OT with finasteride treatment than without it. Moreover, the lowered OT concentration and reduced response to stress in the pregnant sheep, compared to the luteal-phase sheep, may confirm a strong inhibitory influence of brain neurosteroids in this species. An earlier study in rats showed that finasteride administration during late pregnancy resulted in an excessive OT secretory response to interleukin 1b (IL-1b), while AL was found to prevent this reaction. In contrast, in non-pregnant rats treated with AL, the OT response to IL-1b was suppressed. The effects of neurosteroids are thought to be centrally mediated, as AL treatment was shown to decrease Fos expression in virgin rats, whereas finasteride increased Fos expression in late pregnancy in response to systemic IL-1b in both SON and PVN [73]. According to Concas et al. [74], blocking neurosteroid production with a 5α-reductase inhibitor can reduce the brain AL content by approximately 90%. Therefore, the present study indicates that in pregnant ruminants, as in pregnant rats, neurosteroids are heavily involved in the control of OT secretion in both basal and stressful conditions. Since the excitation of OT neurons by various stimuli is inhibited by endogenous opioids during pregnancy [66], it is possible that the interaction between AL and opioids, as suggested by Brunton et al. [73], underlies an adaptive mechanism preventing preterm labor also in this species.

## 5. Conclusions

In conclusion, the present study demonstrated the involvement of neurosteroids in the regulation of OT secretion in sheep. Despite the differential modulatory effects of AL on OT synthesis events in the hypothalamic-neuro-hypophyseal unit of non-pregnant sheep, the neurosteroid markedly inhibited stress-induced OT release. In addition, a strong inhibitory influence of neurosteroids on OT release was found in pregnant sheep, both in normal and stressful conditions. The central effect of neurosteroids in pregnant sheep is consistent with the concept of protecting the fetus from premature birth.

## Figures and Tables

**Table 1:** Sequences of primers used in RT-qPCR.

**Gene**	**Primer**	**Sequence (5′-3′)**	**GeneBank Access No.**
OT	F	GCCTTCTCCCAGCACTGA	X16052
	R	CCTGGGGATGATCAGAGG	
PAM	F	CCCAAAGGTGTTGGATTCAG	XM_004009099
	R	CACCAGAGCAGTCCTTGTGA	
GAPDH	F	GGGTCATCATCTCTGCACCT	NM_001190390.1
	R	GGTCATAAGTCCCTCCACGA	
PPIC	F	TGGAAAAGTCGTGCCCAAGA	XM_004008676.1
	R	TGCTTATACCACCAGTGCCA	

F: forward, R: reverse.

## Data Availability

The data presented in this study are available in this article.
